# Classification and regression model to manage the hospitalization for laparoscopic cholecystectomy

**DOI:** 10.1038/s41598-023-41597-1

**Published:** 2023-09-07

**Authors:** Arianna Scala, Teresa Angela Trunfio, Giovanni Improta

**Affiliations:** 1 Department of Public Health, University of Naples “Federico II”, Naples, Italy; 2 Department of Advanced Biomedical Sciences, University of Naples “Federico II”, Naples, Italy; 3 Interdepartmental Center for Research in Healthcare Management and Innovation in Healthcare (CIRMIS), University of Naples “Federico II”, Naples, Italy

**Keywords:** Biomedical engineering, Health services, Health care

## Abstract

Gallstone disease (GD) is one of the most common morbidities in the world. Laparoscopic Cholecystectomy (LC) is currently the gold standard, performed in about 96% of cases. The most affected groups are the elderly, who generally have higher pre- and post-operative morbidity and mortality rates and longer Length of Stay (LOS). For this reason, several indicators have been defined to improve quality and efficiency and contain costs. In this study, data from patients who underwent LC at the “San Giovanni di Dio e Ruggi d’Aragona” University Hospital of Salerno in the years 2010–2020 were processed using a Multiple Linear Regression (MLR) model and Classification algorithms in order to identify the variables that most influence LOS. The results of the 2352 patients analyzed showed that pre-operative LOS and Age were the independent variables that most affected LOS. In particular, MLR model had a R^2^ value equal to 0.537 and the best classification algorithm, Decision Tree, had an accuracy greater than 83%. In conclusion, both the MLR model and the classification algorithms produced significant results that could provide important support in the management of this healthcare process.

## Introduction

Gallstone disease (GD) is one of the most common diseases in the world, affecting about 18% of the population^1^. Acute cholecystitis results in 90% of cases^2^ from the obstruction of the cystic duct due to the presence of these stones. Laparoscopic Cholecystectomy (LC) is currently the gold standard and has remained so even in the CoViD-19 era^3^ for the management of gallstone disease, performed in about 96% of cases^4^. In fact, compared with Open Cholecystectomy (OA) it offers the advantage of a shorter hospital stay, faster healing and less visible scars^5^. Approximately 3–5% of the LCs, however, are converted to OA during surgery. Risk factors for conversion include age, sex, anatomical features, severity of disease, intraoperative complications (bleeding, internal organs trauma), previous abdominal procedures or the lack of adequate laparoscopic instruments^6,7^. The most affected categories are women and the elderly^8^. The Italian Multicenter Study of Cholelithiasis (MICOL) showed that the overall rate of gallstone disease was 18.8% in women and 9.5% in men^1^. For both sexes, age is the main risk factor for the development of gallstone disease^1,9,10^, with a prevalence of more than 80% in patients older than 90 years^11^. The feasibility of LC in the over-80 population has been evaluated in several studies confirming its safety and efficacy comparable to that obtained in younger patients, making it the most common procedure for this age group^12–15^. The high incidence in this population category intersects with the demographic change in the industrialized countries. In fact, recent years are witnessing a rapid aging of the world population which will lead to an increase in the total prevalence of GD and increased use of the relative surgical procedure^16,17^. Furthermore, it should be considered that this category of patients generally has higher pre- and post-operative morbidity and mortality rates and a longer hospital stay^18^. Today there is a growing interest in improving the quality and efficiency of healthcare processes as much as possible, especially with a view to cost containment. This highlights the need to define and use quality and efficiency indicators^19^. Several tools have been successfully applied to data derived from healthcare processes^20–24^ or to support the management^25–28^. Length of Stay (LOS) is an important performance indicator for hospital costs and management and a key measure of national health system efficiency^29^. Comorbidities, such as heart disease, lung disease and diabetes^30,31^, commonly found in elderly patients, negatively affect this parameter. For example, Valent et al.^32^ showed, through the implementation of regression models, how patients who have diabetes mellitus as a comorbidity have a higher risk of in-hospital death and a longer LOS. Similarly, using a regression model, Ofori-Asenso et al.^33^ showed that the median LOS was 1.1 days longer for patients with a high Charlson comorbidity index (CCI) than for those with a low CCI. These two articles, although in different contexts, validate the use of regression models in the study of LOS and demonstrate the influence of comorbidities. An increase in LOS causes an inevitable increase in costs and a reduction in the number of hospitalizations that can be made^34^. The Italian government, the reference country for this study, has also moved in this direction defining in the National Outcome Plan (in Italian PNE) several indicators that assess the volumes of activity and outcomes of some of the referral health services^35^. For LC, in particular, several indicators were defined to evaluate complications at 30 days and post-operative LOS. After a careful review of the literature, where post-operative LOS varies between 3 and 5 days, the upper limit was set at 3 days. Therefore, it becomes strategic for a hospital to reduce the value of LOS, which is only possible after a full understanding of the main factors that negatively influence it to adopt preventive measures. In order to ensure the national goal, the “San Giovanni di Dio e Ruggi d’Aragona” University Hospital of Salerno (Italy), the target facility of the study, has already analyzed the situation from a Lean Six Sigma perspective^36^ to understand the critical aspects of the process and identify the most appropriate corrective actions.

However, reducing LOS does not only have economic benefits. In fact, many hospitals are successfully performing LC on an outpatient basis, ensuring lower costs and faster turnover^37^. Smith et al.^38^ state that 80% of patients undergoing elective LC can be safely discharged 4–6 h after surgery, with no difference to 23-h monitoring. In fact, the authors make it clear that if no symptoms occur within 3–6 h of surgery without complications, discharge can occur without the need to remain in hospital for days. However, as Topal et al.^39^ show, to make this possible, it is necessary to create specific pathways and define specific patient selection criteria. This new pathway, combined with early LC^40^, can offer significant benefits to the patient, resulting in a high rate of satisfaction and a faster return to normality.

In this study, regression models and classification algorithms were implemented on a dataset consisting of all patients undergoing LC at the “San Giovanni di Dio e Ruggi d’Aragona” University Hospital of Salerno in the years 2010–2020, in order to identify the variables that most influence LOS and to create predictive models useful for application purposes for proper management of hospital resources. This study is an extension of our previous paper, where Multiple Linear Regression (MLR) was applied on a limited set of years and variables^21^.

## Materials and methods

### Data collection

In this study, data from 2352 patients undergoing elective LC at the “San Giovanni di Dio e Ruggi d’Aragona” University Hospital of Salerno in the years 2010–2020 were processed. The information was extracted from the QuaniSDO hospital information system with which the hospital manages and computerizes hospital discharge forms. The dataset was obtained using the inclusion and exclusion criteria provided for the calculation of the indicator “Laparoscopic Cholecystectomy: post-operative hospitalization less than or equal to 3 days” provided by the PNE. The inclusion criteria are as follows:

- LC surgery.
- Primary or secondary diagnosis of gallbladder and bile duct lithiasis.

The exclusion criteria are as follows:

- Patients not resident in Italy.
- Patients younger than 18 years.
- Hospitalizations with a diagnosis of trauma.
- Hospitalization for pregnancy.
- Hospitalization with a diagnosis of malignant tumor of the digestive system.
- Admissions with OA surgery.
- Admissions with patient discharged deceased.
- Admissions in which the patient is transferred from another hospital.
- Admissions for other abdominal surgeries, such as stomach or duodenum/small intestine surgeries, etc.
- Patient undergoing intraoperative cholangiogram or common bile duct exploration.
- Patients had medical admission for another reason.

We decided to exclude cases that generate high LOS variability (such as urgent cases or deceased patients discharged) in order to analyze a more standardized condition, such as elective surgery. The information extracted is the following:

- Gender.
- Age.
- Date of admission.
- Date of surgery.
- Date of discharge.
- Comorbid conditions:
  - Hypertension (yes/no).
  - Diabetes (yes/no).
  - Cardiovascular disease (yes/no).
  - Obesity (yes/no).
  - Allergies (yes/no).
  - Presence of hernia (yes/no).
  - Respiratory disorders (yes/no).
  - Surgery with complications (yes/no).

### Multiple linear regression

MLR is a highly flexible system for analyzing the relationship between one or more independent variables, called predictors, and a single dependent variable, called criterion. At the basis of applying of the model, the dependent variable is assumed to be directly linearly related to the predictors. The relationship describing the model used is the following:

$$\mathrm{y}={\upbeta }_{0}+{\upbeta }_{1}{\mathrm{x}}_{1}+{\upbeta }_{2}{\mathrm{x}}_{2}+{\upbeta }_{3}{\mathrm{x}}_{3}+{\upbeta }_{4}{\mathrm{x}}_{4}+\dots {+\upbeta }_{\mathrm{n}}{\mathrm{x}}_{\mathrm{n}}+e,$$

where y is the dependent variable, x_i_ are the dependent variables which in this case are Gender, Age; Year of Discharge; Comorbid conditions (Hypertension; Diabetes; Cardiovascular disease; Obesity; Allergies; Presence of Hernia; Respiratory Disorders; Surgery with Complications) and pre-operative LOS, the β_i_ values are the coefficients of the model to be determined and e is the error, a random variable. Before implementation, it is necessary to test 6 assumptions that determine the applicability of the linear model, assessing the relationship between variables, the nature of the residuals and the presence of outliers^21,41^. IBM SPSS Statistics Version 26.0 software (IBM Corp, Armonk, NY, USA) was used for model construction and hypothesis testing.

### Classification algorithms

In addition to the construction of the MLR model, Classification algorithms are used both for regression model building and as classifiers. Google Colaboratory (Colab) Cloud Platform was chosen for the implementation. The following algorithms have been implemented as classifiers: Decision Tree (DT), Random Forest (RF), Support Vector Machine (SVM), Naïve Bayes (NB) and Multilayer Perceptron (MLP). These algorithms have been selected for their wide range of applications and excellent results in healthcare^42,43^.

For the study, the dataset was divided into three groups according to the value assumed by the LOS, as follows:

- Group 0: LOS ≤ 3 days.
- Group 1: 4 ≤ LOS ≤ 8 days.
- Group 2: LOS > 8 days.

The choice of thresholds for creating the groups was entirely arbitrary, starting with the threshold of 3 days set by the national indicator. The other values were set to ensure a consistent number of observations for each group. No post-processing techniques were used to balance the dataset. To apply the algorithms, the dataset was divided into training datasets (80% of the total) and test (20% of the total) datasets to calculate evaluation metrics for classification analysis. Both hyperparameter optimization techniques and cross validation were implemented to improve the performance of the algorithms. The scikit-learn library used to implement the classification algorithms makes available to the user the CrossValidator tool that allows the user to partition the dataset into n pairs of separate datasets (training, test) to evaluate a particular set of parameters. The output presented is the average evaluation metric for the models built independently of the particular partitioning done. With the GridSearchCV tool, on the other hand, the hyperparameters of the algorithms are optimized in order to search for the combination that yields the best results for the particular case study. The Table 1 shows the parameters that were arbitrarily selected for the algorithms chosen.

**Table 1:** Selected values of each hyperparameter.

Algorithms	Hyperparameters
SVM	‘kernel’: (‘linear’, ‘rbf’), ‘C’: [1, 10, 100], cv = 10
RF	‘n_estimators’: [5, 10, 15, 20], ‘max_depth’: [2, 5, 7, 9], cv = 10
DT	‘max_depth’: range(3,20), cv = 10
MLP	‘hidden_layer_sizes’: [(50, 50, 50), (50, 100, 50), (100,)], ‘activation’: [‘tanh’, ‘relu’], ‘solver’: [‘sgd’, ‘adam’], ‘alpha’: [0.0001, 0.05],’ learning_rate’: [‘constant’, ’adaptive’], cv = 10
NB	‘var_smoothing’: np.logspace(0,-9, num = 100), cv = 10

Having obtained the best algorithms the Voting Classifier (VC) was implemented. VC uses the prediction of the 5 classifiers to determine the predicted value of each sample through an ensemble technique based on a majority policy. In other words, the sample will be associated with the prediction that receives more than half of the votes, i.e., the predicted value from at least 3 classifiers.

### Ethics approval and consent to participate

The authors declare that all methods were performed in accordance with the Declaration of Helsinki. The institutional review board of “San Giovanni di Dio and Ruggi d’Aragona” University Hospital has approved the study. The institutional review board of “San Giovanni di Dio and Ruggi d’Aragona” University Hospital provided waiver for informed consent for the study. Our data, provided by the Hospital’s Health Department, are completely anonymous and no personal information are linked or linkable to a specific person.

## Results

**Figure 1:**
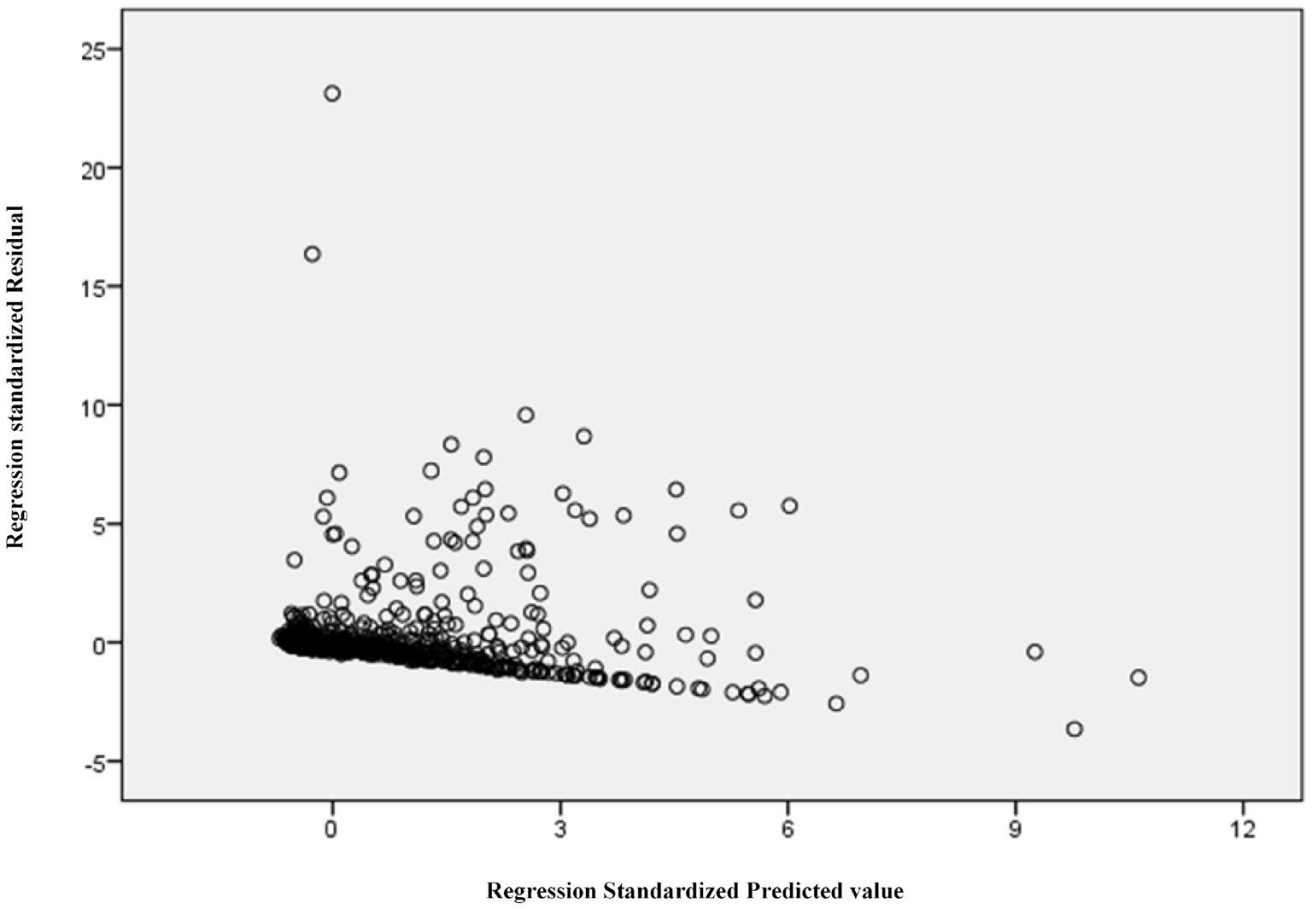
Standardized residual regression vs the regression standardized predicted value.

First, data from the 2352 patients were analyzed using the MLR model. Before implementing the model, however, it was necessary to verify the consistency of the six hypotheses defined in the previous paragraph. After ensuring the linearity of the relationship between independent variables and dependent variable, Tolerance and VIF were calculated. In all cases, a Tolerance value greater than 0.2 and a VIF value less than 10 was obtained, values that guarantee the absence of multicollinearity. Assumptions 3 and 4 on the residuals are determined in graphical form. Figure 1 shows the graph of the standardized residual regression vs the regression standardized predicted value.

It can be seen from the Figure that the residuals are distributed around 0, which suggests that the homoscedasticity assumption is not violated.

On the other hand, the absence of outliers is ensured by a Cook’s distance less than 1 for each record. Finally, the independence of the residues is demonstrated by the Durbin-Watson test result within the acceptability range of 0.0 and 4.0^44^. At this point, the MLR was implemented. Table 2 shows the results of the regression model and Durbin–Watson test.

**Table 2:** Model summary.

	R	R-squared	Adjusted R-squared	Std. error	Durbin–Watson test
Regression model	0.733	0.537	0.535	13.029	1.823

The value of R^2^ greater than 0.5 showed that the model was robust enough for this particular type of application^45^. Table 3 shows the value of the coefficients βi, the t-test and the p-value obtained for the independent variables considered. The test is considered verified when p-value is less than 0.05.

**Table 3:** Regression coefficients and results of t-test.

	Unstandardized coefficients	Std. error	Standardised coefficients	t	p-value
Intercept	295.887	191.269	–	1.547	0.122
Year of discharge	− 0.145	0.095	− 0.022	− 1.530	0.126
Gender	0.206	0.553	0.005	0.373	0.709
Age	− 0.014	0.019	− 0.011	− 0.760	0.448
Pre-operative LOS	1.471	0.029	0.736	51.394	**0.000**
Hypertension	0.434	0.813	0.008	0.533	0.594
Diabetes	1.891	1.319	0.021	1.434	0.152
Obesity	− 1.172	1.507	− 0.011	− 0.778	0.437
Cardiovascular disease	− 0.551	1.322	− 0.006	− 0.417	0.677
Allergies	− 1.521	1.940	− 0.011	− 0.784	0.433
Presence of hernia	− 0.641	2.231	− 0.004	− 0.287	0.774
Respiratory disorders	− 0.623	2.056	− 0.004	− 0.303	0.762
Surgery with complications	− 0.781	0.855	− 0.014	− 0.913	0.361

Significant values are in bold.

Observing the p-value column, the only variable that significantly affected the LOS was the pre-operative LOS. Therefore, the selected classification algorithms were implemented. Before proceeding, it was necessary to arbitrarily partition the LOS into classes. The baseline characteristics of the 3 Groups are shown in Table 4.

**Table 4:** Baseline characteristics.

	LOS			p-value
	Group 0 N = 1013	Group 1 N = 778	Group 2 N = 561	
Year of discharge				**0.000**
2010	13	24	9	
2011	84	76	31	
2012	79	72	43	
2013	87	75	50	
2014	74	112	41	
2015	49	101	55	
2016	80	90	55	
2017	153	42	99	
2018	125	60	86	
2019	149	74	61	
2020	120	52	31	
Gender				0.479
Male	397	324	234	
Female	616	454	327	
Age				**0.000**
Mean ± STD	50.41 ± 14.56	52.78 ± 15.06	56.20 ± 16.27	
Pre-operative LOS				**0.000**
Mean ± STD	0.44 ± 0.53	1.75 ± 1.34	16.68 ± 13.85	
Hypertension				**0.000**
0	885	653	448	
1	128	125	113	
Diabetes				**0.027**
0	978	736	526	
1	35	42	35	
Obesity				0.221
0	974	759	540	
1	39	19	21	
Cardiovascular disease				**0.000**
0	983	736	513	
1	30	42	48	
Allergies				0.197
0	990	760	555	
1	23	18	6	
Presence of hernia				0.863
0	997	766	554	
1	16	12	7	
Respiratory disorders				0.002
0	1002	765	541	
1	11	13	20	
Surgery with complications				**0.000**
0	962	678	399	
1	51	100	162	

Significant values are in bold.

The classes showed significant differences not only in pre-operative LOS but also in the presence of complications during surgery, in the Age and in some cardiovascular-related comorbidities. Before presenting the results, the hyperparameters of each algorithm obtained as a result of the optimization process are shown in Table 5.

**Table 5:** Best parameters.

Algorithm	Best parameters
DT	{‘max_depth’: 4}
RF	{‘max_depth’: 9, ‘n_estimators’: 15}
SVM	{‘C’: 10, ‘kernel’: ‘linear’}
NB	{var_smoothing = 0.0006}
MLP	{‘activation’: ‘tanh’, ‘alpha’: 0.0001, ‘hidden_layer_sizes’: (50, 50, 50), ‘learning_rate’: ‘adaptive’, ‘solver’: ‘adam’}
Voter	{‘voting’: ‘hard’}

Table 6 reports the results obtained in terms of accuracy, precision, recall and F-measure.

**Table 6:** Performance metrics of all selected algorithms.

Performance metrics	Class	DT	RF	SVM	NB	MLP
Accuracy (%)	Overall	0.83	0.81	0.80	0.74	0.74
Precision (%)	1	0.85	0.81	0.78	0.65	0.64
	2	0.76	0.75	0.75	0.81	0.76
	3	0.93	0.91	0.91	0.92	0.99
Recall (%)	1	0.82	0.83	0.83	0.96	0.96
	2	0.81	0.75	0.71	0.41	0.44
	3	0.88	0.88	0.89	0.89	0.82
F-measure (%)	1	0.83	0.82	0.81	0.78	0.77
	2	0.78	0.75	0.73	0.54	0.56
	3	0.90	0.89	0.90	0.90	0.89

With an accuracy of 83.0% DT had the best performance, followed by RF with an accuracy of 81.0%, SVM with an accuracy of 80.0% and finally NB and MLP with an accuracy of 74.0%. For all algorithms, the worst results are obtained in the classification of the second class, i.e. patients with a post-operative LOS between 4 and 8 days. The best results, however, when considering F-measure are recorded for the third class, at prolonged LOS, which is the one of most interest to health care management. Details of the classification for the best algorithm are shown in Table 7.

**Table 7:** Decision tree confusion matrix.

Real/predicted	1	2	3
1	173	34	0
2	22	134	4
3	1	11	92

**Figure 2:**
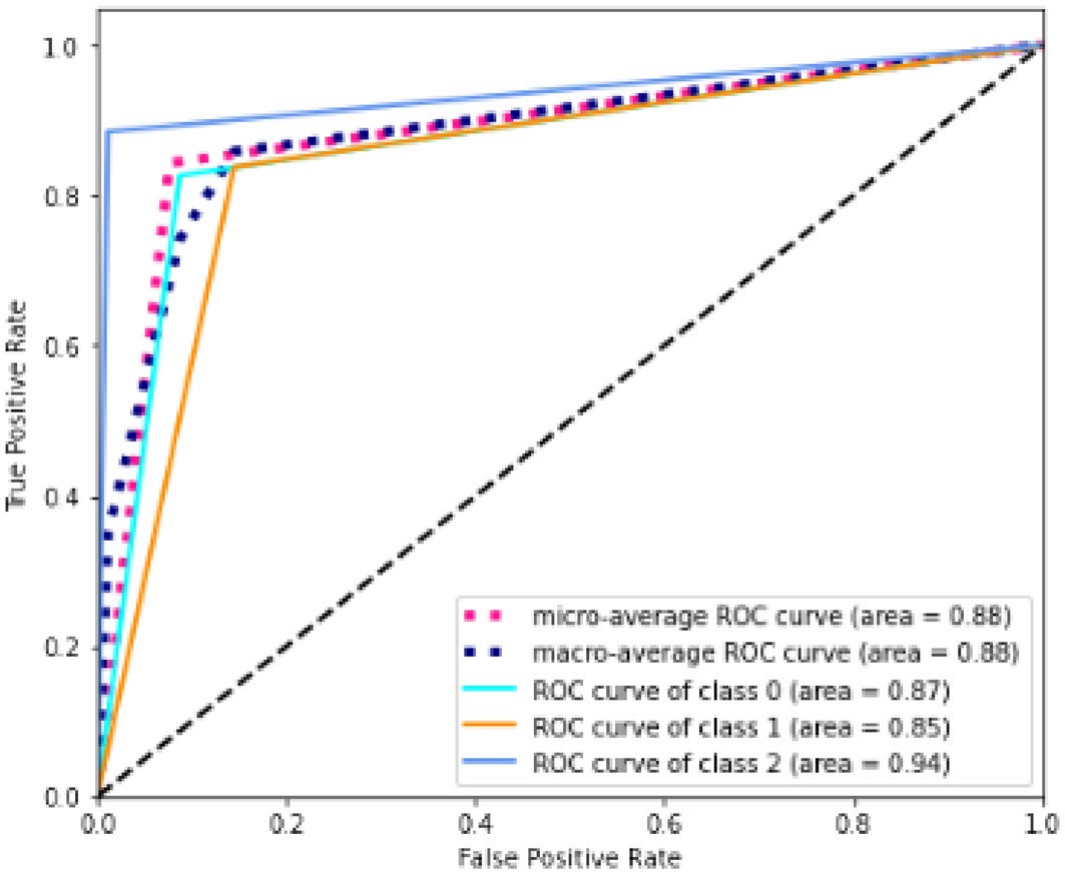
ROC curves.

Figure 2 shows the ROC curves for DT.

**Figure 3:**
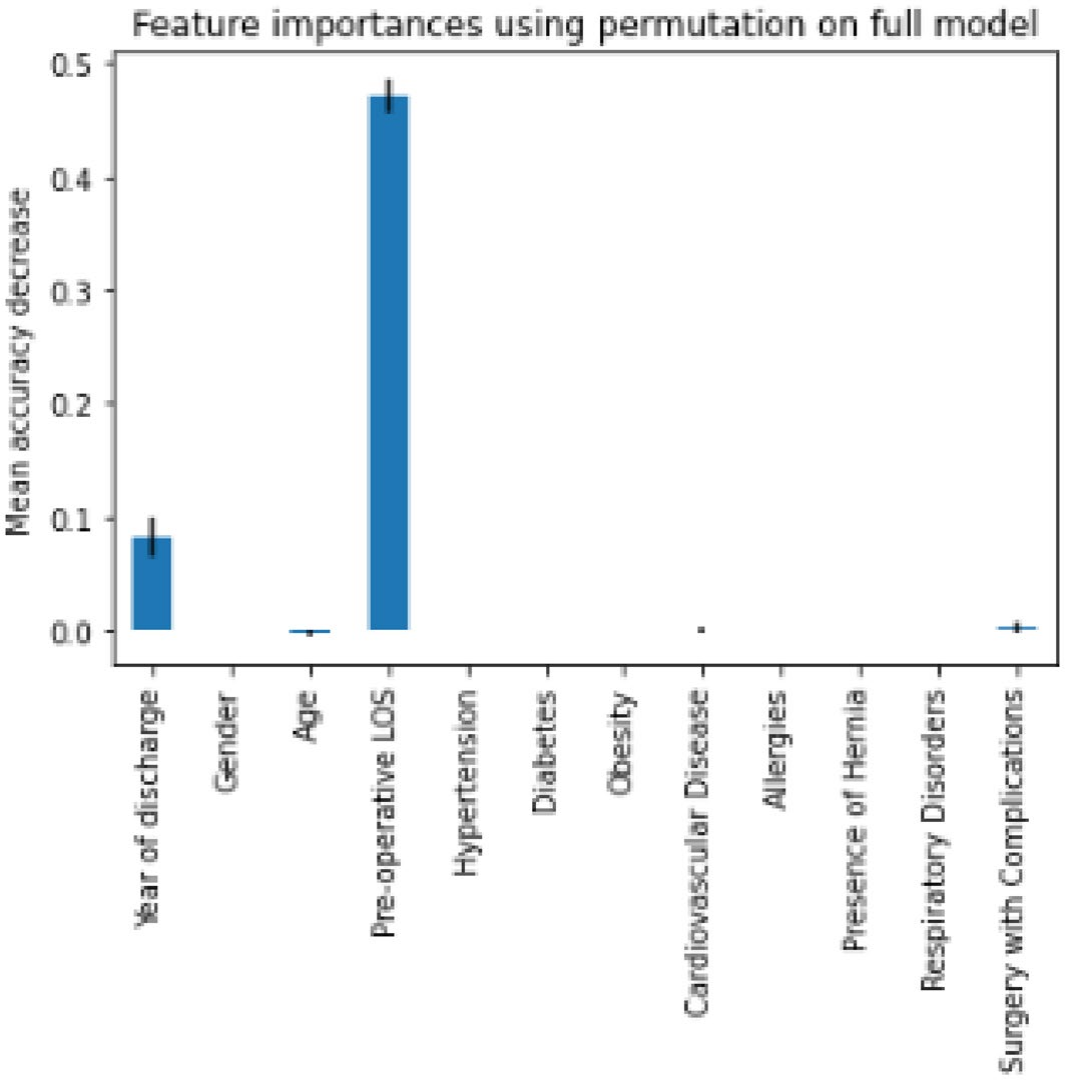
Permutation feature importance.

It can be seen from the figure that the largest area compared to the “no benefit” line (black discontinuous line) is obtained for the third class for which the algorithm returned the best results. On average, the area obtained still reaches a significant result of 0.88. Figure 3 reports the results of Permutation Feature Importance.

Permutation feature importance allows visualization among the independent variables of which ones most influence the model by going to measure performance using a corrupted version of one of the variables each time. Figure shows that pre-operative LOS was one of the variables that reasonably influences the output, followed by the Year of Discharge. Age and complications incurring during surgery, on the other hand, had little effect. Finally, the VC has been implemented. The Accuracy achieved by a ‘Hard’ voting technique that classifies the input data according to the mode of all predictions of the various classifiers reaches 84.0% improving by 1.0% the value achieved by DT.

## Discussion

In this study, the LOS patients who underwent LC was analyzed. Specifically, starting from the computerized hospital discharge forms, Age, Gender, Presence of Comorbidities, Date of Admission, Date of Discharge and the Date of LC procedure were extracted for each patient. From the date variables, it was possible to calculate the pre-operative LOS used, together with the others, as an independent variable. To allow the facility to test how the patient clinical and demographic variables affected LOS, a MLR model was built and classification algorithms were implemented. In the first case, the obtained MLR model had an R^2^ equal to 0.537, which demonstrated the robustness of the model in making predictions in this particular application. The results of the statistical test showed that the variable that significantly affected LOS was only the pre-operative LOS, obvious result being the pre-operative LOS a part of the total LOS. This result becomes indicative when considering that the procedures analysed are all carried out as electives. The preoperative phase thus showed a high variability, which could be limited by prehospitalisation. Then, the classification algorithms were implemented. For this purpose, the patients were divided into three groups according to the value assumed by the LOS (< 3 days, 4–8 days, > 8 days). The best algorithms with an Accuracy of 83.0% was DT, followed by RF, SVM, MLP and NB. Class 1, consisting of all patients with a LOS between 4 and 8 days, was the one for which the worst results are obtained due to a reduced number of entries. On the other hand, the best results were obtained on Class 2, which is the one that includes patients with prolonged LOS and on whom health care management needs to focus more for waste reduction. Permutation feature importance applied to the best algorithm made it possible to identify which of the variables considered has a significant effect in classification. In addition to pre-operative LOS, already highlighted by the MLR model, Year of discharge, Age and the complications incurring during surgery also had an effect, although minimal, on the output. In fact, over the years there has been increasing use of the laparoscopic procedure even on older patients demonstrating significant benefits^46^. Older patients, however, have shown prolonged hospital stay in several studies. In particular, Chang et al.^47^ showed that patients over 80 years old had a significantly longer length of perioperative hospital stay, while those over 65 generally record higher risk of having minor complications. The same result is also confirmed by Firilas et al.^48^ who although not noting differences in complication rates between patients aged 65–75 and patients over 75 years, highlight how younger patients had a significantly shorter mean length of hospitalization. Complications during surgery, which in severe cases can lead to conversion to an OA, can result in a longer hospital stay and greater risk for the patient. This condition has been studied as shown previously for the older population, which generally presents with worse clinical conditions that could lead to more complicated surgery^49^. The dependence on the year of discharge can be easily explained by taking into account a previous study of ours showing the interventions that were put in place in Lean Six Sigma logic to reduce the LOS^36^. Finally, the voting technique further improved the final performance, achieving an accuracy of 84.0%.

Although a single model was created and new variables added, we could not replicate the excellent results obtained with the comparison in terms of the MLR model before and after implementation of corrective actions to reduce postoperative LOS done in a previous study^21^.

The clinical implications of this study are intended primarily in healthcare planning and programming activities. Knowing the variables that most impact inpatient stays as well as building predictive models that help determine the value of LOS a priori could support both the identification of corrective actions—such as a pre-hospitalization phase or an increase in operating sessions^36^—for process optimization but also bed management activities and waiting list management.

The limitations of the study are multiple. The comparative analysis of the model does include the need for re-intervention, conversion to OA, the degree of complexity of comorbidities considered and the presence of confounding factors, such as infections. These limits are mainly related to the source of data extraction, the hospital discharge form, which does not allow a clear clinical picture to be made. In addition, although the study includes a significant number of years of observation and patients, the fact that it is a single-center study does not allow the results obtained to be generalizable. From a methodological point of view, for the implementation of the classification algorithms we made an arbitrary subdivision not based on scientific evidence and the inclusion of Pre-Operative LOS may have obscured the effect of some comorbidities. Finally, the effects of CoViD-19 on the complexity of the treated cases are not taken into consideration^50^.

## Conclusion

In this study, Age, Gender, Pre-operative LOS and Presence of selected Comorbidities were used as independent variables in the construction of an MLR model and classification algorithms in order to predict the LOS of patients undergoing LC at the “San Giovanni di Dio and Ruggi d’Aragona” University Hospital of Salerno (Italy) in the years 2010–2020. The obtained MLR model and implemented algorithms have been shown to be valid in predicting LC hospitalization as well as in identifying the variables that have a more significant effect among those considered.

Future developments in the work include expanding the independent variables provided as input to the model and the number of patients should include in the study to improve the performance of the algorithms and provide the most accurate tool possible.

## Data Availability

The datasets generated and/or analyzed during the current study are not publicly available for privacy reasons but could be made available from the corresponding author on reasonable request.

## Abbreviations

LOS: Length of Stay
LC: Laparoscopic cholecystectomy
OA: Open cholecystectomy
MLR: Multiple linear regression
RF: Random Forest
DT: Decision tree
SVM: Support vector machine
MLP: Multilayer perception
NB: Naive Bayes
VC: Voting Classifier

## References

[1] Festi D, Dormi A, Capodicasa S, Staniscia T, Attili AF, Loria P, Pazzi P, Mazzella G, Sama C, Roda E, Colecchia A (2008). Incidence of gallstone disease in Italy: Results from a multicenter, population-based Italian study (the MICOL project). World J. Gastroenterol..

[2] Strasberg SM (2008). Acute calculous cholecystitis. N. Engl. J. Med..

[3] Campanile FC, Podda M, Arezzo A (2020). Acute cholecystitis during COVID-19 pandemic: A multisocietary position statement. World J. Emerg. Surg..

[4] Berthou JCh, Dron B, Charbonneau P, Moussalier K, Pellissier L (2007). Evaluation of laparoscopic treatment of common bile duct stones in a prospective series of 505 patients: Indications and results. Surg. Endosc..

[5] Kuwabara K, Matsuda S, Ishikawa KB, Horiguchi H, Fujimori K (2010). Comparative quality of laparoscopic and open cholecystectomy in the elderly using propensity score matching analysis. Gastroenterol. Res. Pract..

[6] Bhama AR (2015). Factors associated with conversion from laparoscopic to open colectomy using the National Surgical Quality Improvement Program (NSQIP) database. Colorectal Dis..

[7] Tan PY (2008). Laparoscopically assisted colectomy: A study of risk factors and predictors of open conversion. Surg. Endosc..

[8] Saia M, Mantoan D, Buja A (2013). Time trend and variability of open versus laparoscopic cholecystectomy in patients with symptomatic gallstone disease. Surg. Endosc..

[9] Kuy S, Sosa JA, Roman SA, Desai R, Rosenthal RA (2011). Age matters: A study of clinical and economic outcomes following cholecystectomy in elderly Americans. Am. J. Surg..

[10] Parmar AD (2014). PREOP-gallstones: A prognostic nomogram for the management of symptomatic cholelithiasis in older patients. Ann. Surg..

[11] Cheng SP, Chang YC, Liu CL (2008). Factors associated with prolonged stay after laparoscopic cholecystectomy in elderly patients. Surg. Endosc..

[12] Dubecz A (2012). Cholecystectomy in the very elderly-is 90 the new 70?. J. Gastrointest. Surg..

[13] Ambe PC, Weber SA, Christ H, Wassenberg D (2015). Primary cholecystectomy is feasible in elderly patients with acute cholecystitis. Aging Clin. Exp. Res..

[14] Kakucs T, Harsanyi L, Kupcsulik P, Lukovich P (2016). The role of laparoscopy in cholecystectomy in patients with age of 80 and above. Orv. Hetil..

[15] Pålsson S, Saliba G, Sandblom G (2016). Outcome after cholecystectomy in the elderly: A population-based register study. Scand. J. Gastroenterol..

[16] Das, R. A. Silver Tsunami invades the health of nations—forbes. *Forbes Magazine*. http://www.forbes.com/sites/reenitadas/2015/08/11/a-silver-tsunami-invades-the-health-of-nations/#6b202ba34c59 (2015).

[17] Bartels SJ, Naslund JA (2013). The underside of the silver tsunami—Older adults and mental health care. N. Engl. J. Med..

[18] Kuy S, Sosa JA, Roman SA, Desai R, Rosenthal RA (2011). Age matters: A study of clinical and economic outcomes following cholecystectomy in elderly Americans. Am. J. Surg..

[19] Jiménez RE, Lam RM, Marot M (2004). Observed-predicted length of stay for an acute psychiatric department, as an indicator of inpatient care inefficiencies. Retrospective case-series study. BMC Health Serv. Res..

[20] Trunfio TA, Borrelli A, Improta G (2022). Implementation of predictive algorithms for the study of the endarterectomy LOS. Bioengineering.

[21] Scala, A. *et al*. Modelling the hospital length of stay for patients undergoing laparoscopic cholecystectomy through a multiple regression model. In *2021 5th International Conference on Medical and Health Informatics (ICMHI 2021)* 68–72. 10.1145/3472813.3472826 (Association for Computing Machinery, 2021).

[22] Raiola E (2020). Implementation of lean practices to reduce healthcare associated infections. Int. J. Healthc. Technol. Manag..

[23] Improta G (2020). Fuzzy logic-based clinical decision support system for the evaluation of renal function in post-transplant patients. J. Eval. Clin. Pract..

[24] Ponsiglione AM, Cosentino C, Cesarelli G, Amato F, Romano M (2021). A comprehensive review of techniques for processing and analyzing fetal heart rate signals. Sensors.

[25] Improta G. *et al*. Management of the diabetic patient in the diagnostic care pathway. In *8th European Medical and Biological Engineering Conference. EMBEC 2020. IFMBE Proceedings*, Vol. 80 (eds. Jarm, T. *et al.*). 10.1007/978-3-030-64610-3_88 (Springer, 2021).

[26] Cesarelli G (2021). An innovative business model for a multi-echelon supply chain inventory management pattern. J. Phys. Conf. Ser..

[27] Cortesi PA, Castaman G, Trifirò G, Creazzola SS, Improta G, Mazzaglia G (2019). Cost-effectiveness and budget impact of emicizumab prophylaxis in haemophilia a patients with inhibitors. Thromb. Haemost..

[28] Bonavolontà P (2019). Postoperative complications after removal of pleomorphic adenoma from the parotid gland: A long-term follow up of 297 patients from 2002 to 2016 and a review of publications. Br. J. Oral Maxillofac. Surg..

[29] Kulinskaya E, Kornbrot D, Gao H (2005). Length of stay as a performance indicator: Robust statistical methodology. IMA J. Manag. Math..

[30] Extermann M (2000). Measuring comorbidity in older cancer patients. Eur. J. Cancer.

[31] Extermann M (2000). Measurement and impact of comorbidity in older cancer patients. Crit. Rev. Oncol. Hematol..

[32] Valent F, Tonutti L, Grimaldi F (2017). Does diabetes mellitus comorbidity affect in-hospital mortality and length of stay? Analysis of administrative data in an Italian Academic Hospital. Acta Diabetol..

[33] Ofori-Asenso R, Zomer E, Chin KL, Si S, Markey P, Tacey M, Curtis AJ, Zoungas S, Liew D (2018). Effect of comorbidity assessed by the Charlson comorbidity index on the length of stay, costs and mortality among older adults hospitalised for acute stroke. Int. J. Environ. Res. Public Health.

[34] Thiele RH (2015). Standardization of care: Impact of an enhanced recovery protocol on length of stay, complications, and direct costs after colorectal surgery. J. Am. Coll. Surg..

[35] Italian Ministry of Health, National Agency for Regional Health Services (AGENAS) (2020). PNE Programma Nazionale Esiti—Edizione 2020.

[36] Trunfio, T. A. *et al*. Application of the Lean Six Sigma approach to the study of the LOS of patients who undergo laparoscopic cholecystectomy at the San Giovanni di Dio and Ruggi d’Aragona University Hospital. In *2021 5th International Conference on Medical and Health Informatics (ICMHI 2021)* 50–54. 10.1145/3472813.3472823 (Association for Computing Machinery, 2021).

[37] Ji W (2010). Outpatient versus inpatient laparoscopic cholecystectomy: A single center clinical analysis. Hepatobiliary Pancreat. Dis. Int..

[38] MarkSmith II, Wheeler W, Ulmer MB (1997). Comparison of outpatient laparoscopic cholecystectomy in a private nonteaching hospital versus a private teaching community hospital. J. Soc. Laparoendosc. Surg..

[39] Topal B (2007). Outpatient laparoscopic cholecystectomy: Clinical pathway implementation is efficient and cost effective and increases hospital bed capacity. Surg. Endosc..

[40] Casillas RA, Yegiyants S, CraigCollins J (2008). Early laparoscopic cholecystectomy is the preferred management of acute cholecystitis. Arch. Surg..

[41] Scala A, Borrelli A, Improta G (2022). Predictive analysis of lower limb fractures in the orthopedic complex operative unit using artificial intelligence: The case study of AOU Ruggi. Sci. Rep..

[42] Ponsiglione AM (2023). predictive analysis of hospital stay after caesarean section: A single-center study. Bioengineering.

[43] Scala A (2022). Risk factors analysis of surgical infection using artificial intelligence: A single center study. Int. J. Environ. Res. Public Health.

[44] Field, A. *Discovering Statistics Using spss Third Edition* (2009).

[45] Hamilton DF, Ghert M, Simpson AHRW (2015). Interpreting regression models in clinical outcome studies. Bone Jt. Res..

[46] Dua A (2014). National trends in the adoption of laparoscopic cholecystectomy over 7 years in the United States and impact of laparoscopic approaches stratified by age. Minim. Invas. Surg..

[47] Chang W-T (2009). Laparoscopic cholecystectomy in aged patients. Hepatogastroenterology.

[48] Firilas A, Duke BE, Max MH (1996). Laparoscopic cholecystectomy in the elderly. Surg. Endosc..

[49] Tucker JJ (2011). Laparoscopic cholecystectomy is safe but underused in the elderly. Am. Surg..

[50] Pati-Alam A (2021). P-EGS21 the impact of COVID-19 on operative difficulty and outcomes of laparoscopic cholecystectomy. Br. J. Surg..

